# α-Synuclein-Induced Synapse Damage in Cultured Neurons Is Mediated by Cholesterol-Sensitive Activation of Cytoplasmic Phospholipase A_2_

**DOI:** 10.3390/biom5010178

**Published:** 2015-03-09

**Authors:** Clive Bate, Alun Williams

**Affiliations:** 1 Department of Pathology and Pathogen Biology, Royal Veterinary College, Hawkshead Lane, North Mymms, Herts AL9 7TA, UK; 2 Department of Veterinary Medicine, University of Cambridge, Madingley Road, Cambridge CB3 OES, UK; E-Mail: aw510@cam.ac.uk

**Keywords:** alpha-synuclein, cholesterol, Parkinson’s disease, phospholipase A_2_, platelet-activating factor, synapse, synaptophysin

## Abstract

The accumulation of aggregated forms of the α-synuclein (αSN) is associated with the pathogenesis of Parkinson’s disease (PD) and Dementia with Lewy Bodies. The loss of synapses is an important event in the pathogenesis of these diseases. Here we show that aggregated recombinant human αSN, but not βSN, triggered synapse damage in cultured neurons as measured by the loss of synaptic proteins. Pre-treatment with the selective cytoplasmic phospholipase A_2_ (cPLA_2_) inhibitors AACOCF_3_ and MAFP protected neurons against αSN-induced synapse damage. Synapse damage was associated with the αSN-induced activation of synaptic cPLA_2_ and the production of prostaglandin E_2_. The activation of cPLA_2_ is the first step in the generation of platelet-activating factor (PAF) and PAF receptor antagonists (ginkgolide B or Hexa-PAF) also protect neurons against αSN-induced synapse damage. αSN-induced synapse damage was also reduced in neurons pre-treated with the cholesterol synthesis inhibitor (squalestatin). These results are consistent with the hypothesis that αSN triggered synapse damage via hyperactivation of cPLA_2_. They also indicate that αSN-induced activation of cPLA_2_ is influenced by the cholesterol content of membranes. Inhibitors of this pathway that can cross the blood brain barrier may protect against the synapse damage seen during PD.

## 1. Introduction

Parkinson’s disease (PD) is a major neurodegenerative motor disorder which affects approximately 2% of the population over 65. In addition to the defining characteristics of bradykinesia, resting tremor and rigidity, there significant psychiatric and autonomic symptoms are also observed in a large % of patients [1]. The most common of the non-motor symptoms is Parkinson’s disease dementia (PDD), with a cumulative prevalence up to 75% of cases [2]. Also observed in PD patients is Dementia with Lewy Bodies, a similar condition to PDD in which dementia, rather than motor symptoms, are the primary clinical manifestations. Dementia with Lewy Bodies is the second most common cause of dementia after Alzheimer’s disease and is characterized by progressive cognitive decline and parkinsonism [3]. Currently, treatment is based upon the alleviation of symptoms as there is no long-term cure for PD, PDD or Dementia with Lewy Bodies.

The alpha-synuclein (αSN) positive intraneuronal inclusion known as a Lewy body is a major histopathological feature of PD, PDD and Dementia with Lewy Bodies. While the presence of Lewy bodies in the substantia nigra is diagnostic for PD, aggregated αSN is also seen in many non-nigral regions and consequently may account for the wide range of non-motor symptoms. The molecular mechanisms underlying the pathological changes in PD are poorly understood but aggregated forms of αSN are thought to play a central role. αSN is predominantly expressed in central nervous system neurons where it is found within pre-synaptic terminals where it regulates synaptic vesicle formation and neurotransmitter release [4,5] and can affect synaptic plasticity and hence learning [6]. Recent evidence suggests that aggregates of αSN accumulate at the pre-synaptic membrane and trigger synapse damage in PD and Dementia with Lewy Bodies [7,8,9]. Furthermore, the transfer of αSN aggregates to adjacent neurons [10,11] may account for the progression of αSN pathology through the brain in a manner that is similar to the staging of tau pathology in Alzheimer’s disease [12]. Thus, synapse damage in the hippocampus is characteristic of the PD patients that develop dementia [13] and in a rat model of α-synucleinopathy, synaptic damage preceded neuronal loss [14]. Thus, synapse damage is a common feature observed in PD, PDD and Dementia with Lewy Bodies.

The molecular mechanisms that underlie αSN aggregate-induced synapse damage are not understood. Such processes have been examined by incubating cultured neurons with αSN aggregates [15] in a similar manner to how amyloid-β (Aβ) peptides are used to investigate the pathogenesis of Alzheimer’s disease. An understanding of the molecular mechanisms that underlie synapse damage caused by aggregated αSN may help identify drugs that reduce this process. To investigate these mechanisms the effect of αSN aggregates on cultured cortical neurons was studied; synaptic density was measured by quantifying the amounts of synaptophysin and cysteine string protein (CSP) using ELISAs [16]. The loss of synaptic proteins including synaptophysin shows a close correlation with cognitive decline in Alzheimer’s disease [17,18] and reflects the loss of other synaptic proteins from neuronal cultures [19]. In this study we used cultured neurons to explore the role of specific cell signaling pathways and cholesterol in neuronal responses to aggregated αSN.

## 2. Results

### 2.1. αSN Triggered the Loss of Synaptic Proteins from Cultured Neurons

**Figure 1:**
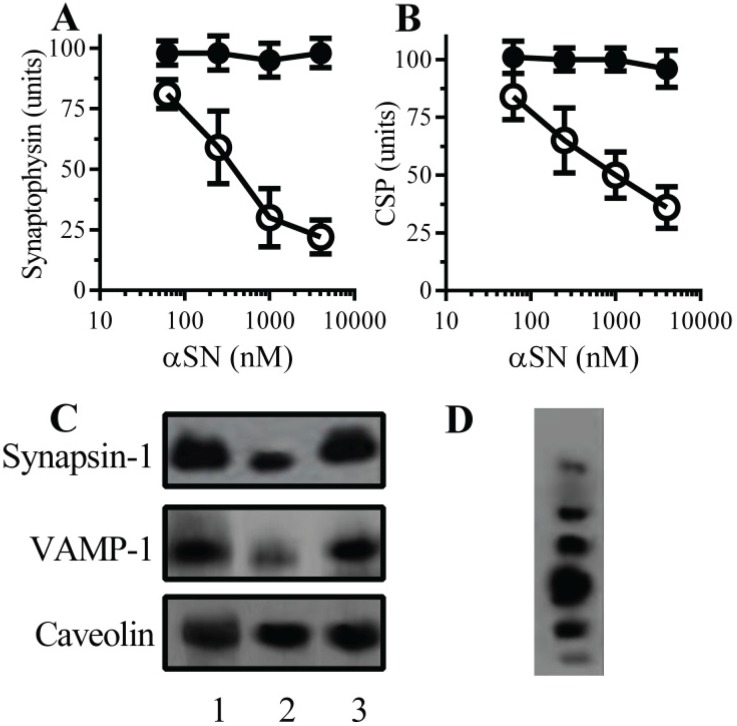
α-synuclein (αSN) triggered the loss of synaptic proteins from neurons—The amounts of synaptophysin (**A**) and cysteine string protein (CSP) (**B**) in neurons incubated with αSN (○) or βSN (●) as shown. Values are means ± SD from triplicate experiments performed three times, *n* = 9; (**C**) Immunoblots showing the amounts of synapsin-1, vesicle-associated membrane protein (VAMP)-1 and caveolin in neurons incubated with control medium (1), 1 µM αSN (2), 1 µM βSN (3); (**D**) Immunoblot showing aggregates of recombinant human αSN separated by non-denaturing polyacrylamide gel electrophoresis (PAGE).

The synapse damage seen in PD and Dementia with Lewy Bodies is closely associated with αSN oligomers [7,8,9]. In an *in vitro* model the addition of aggregated recombinant human αSN, but not βSN, caused a dose-dependent reduction in synaptic proteins including synaptophysin [15] (Figure 1A) and CSP (Figure 1B) from cultured neurons. Immunoblots showed that the loss of synaptophysin and CSP from cultured neurons was accompanied by the loss of other synaptic proteins including synapsin-1 and vesicle-associated membrane protein (VAMP)-1 (Figure 1C). Incubation with αSN did not affect the amounts of caveolin in neuronal cultures, nor did it significantly reduce cell viability as measured by the thiazolyl blue tetrazolium (MTT) method, indicating that there was no significant neuronal death in these cultures (98% cell viability ± 6 compared with 100% ± 5, *n* = 9, *p* = 0.43).

### 2.2. PLA_2_ Inhibitors Protect Neurons against αSN-Induced Synapse Damage

**Figure 2:**
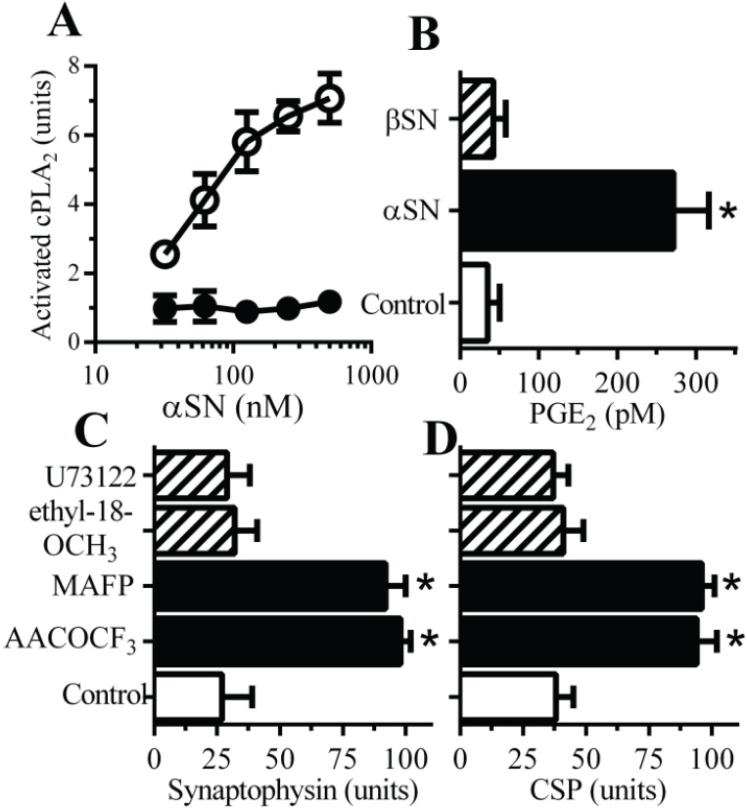
PLA_2_ inhibitors protect neurons against αSN-induced synapse damage—(**A**) The amounts of activated cPLA_2_ in synaptosomes incubated with αSN (○) or βSN (●) as shown. Values are means ± SD from triplicate experiments performed three times, *n* = 9; (**B**) The concentrations of PGE_2_ produced by synaptosomes incubated with control medium (□), 500 nM αSN (■) or 500 nM βSN (striped bar). Values are means ± SD from triplicate experiments performed three times, *n* = 9. * = significantly higher than in control synaptosomes. The amounts of synaptophysin (**C**) and CSP (**D**) in neurons pre-treated with control medium (□), phospholipase A_2_ inhibitors (1 µM AACOCF_3_ or 1 µM MAFP) (■) or phospholipase C inhibitors (10 µM U73122 or 10 µM ethyl-18-OCH_3_) (striped bars) and incubated with 1 μM αSN. Values are means ± SD from triplicate experiments performed four times, *n* = 12. * = significantly higher than in control neurons incubated with αSN.

Prior studies showed that synapse damage induced by prion peptides or amyloid-β (Aβ), thought to be the causative agent in the pathogenesis of Alzheimer’s disease, was associated with activation of synaptic cytoplasmic phospholipase A_2_ (cPLA_2_) [16]. Here we show that αSN, but not βSN, caused a dose-dependent activation of cPLA_2_ in synaptosomes (Figure 2A). The activation of cPLA_2_ was accompanied by the release of prostaglandin (PG)E_2_ (Figure 2B). The synaptophysin content of neurons was not significantly affected by treatment with cPLA_2_ inhibitors, 5 µM arachidonyl trifluoromethyl ketone (AACOCF_3_) (100 units synaptophysin ± 4 compared to 101 ± 4, *n* = 12, *p* = 0.5) or 5 µM methyl arachidonyl fluorophosphonate (MAFP) (100 ± 4 *vs.* 102 ± 6, *n* = 12, *p* = 0.4) showing that these drugs did not stimulate synaptogenesis, nor did they damage synapses. In primary cultures pre-treatment with either 1 µM AACOCF_3_ or 1 µM MAFP protected neurons against the αSN-induced reductions in synaptophysin (Figure 2C) and CSP (Figure 2D). In contrast, pre-treatment with phospholipase C inhibitors (10 µM U73122 or ethyl-18-OCH_3_) did not affect αSN-induced reductions in synaptophysin and CSP. Collectively these results support the hypothesis that hyperactivation of cPLA_2_ is involved in involved in αSN-induced synapse damage.

### 2.3. Cyclooxygenase Inhibitors Protect Neurons against αSN-Induced Synapse Damage

**Figure 3:**
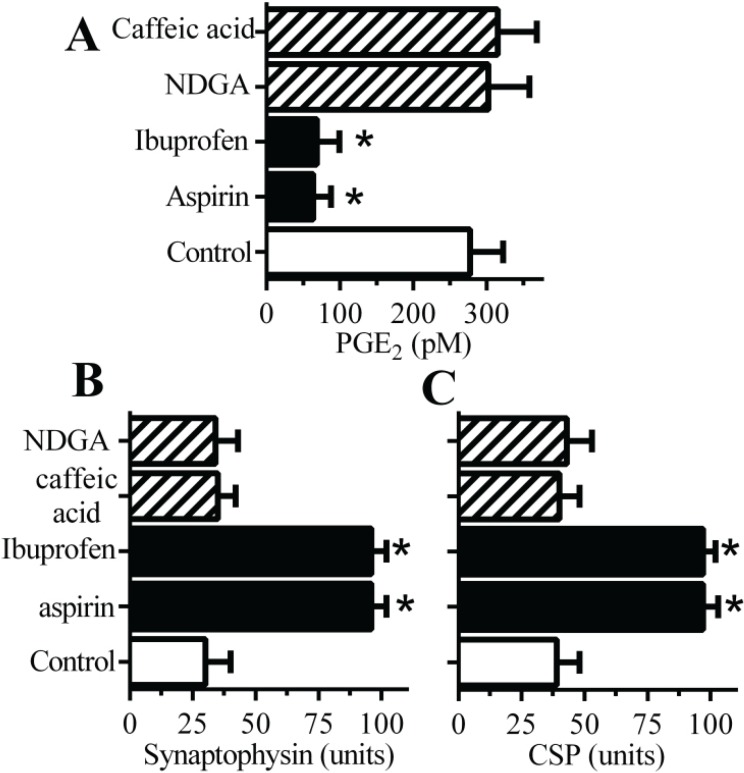
Cyclooxygenase inhibitors protect neurons against αSN-induced synapse damage. (**A**) The concentrations of PGE_2_ produced by synaptosomes pre-treated with a vehicle control (□), cyclooxygenase inhibitors (aspirin and ibuprofen) (■) or lipoxygenase inhibitors (caffeic acid and nordihydroguaiaretic acid (NDGA)) (striped bars) and incubated with 500 nM αSN. Values are means ± SD from triplicate experiments performed three times, *n* = 9. * = significantly lower than control synaptosomes incubated with 500 nM αSN. The amounts of synaptophysin (**B**) and CSP (**C**) in neurons pre-treated with a vehicle control (□), cyclooxygenase inhibitors (aspirin and ibuprofen) (■) or lipoxygenase inhibitors (caffeic acid and NDGA) (striped bars) and incubated with 1 μM αSN. Values are means ± SD from triplicate experiments performed four times, *n* = 12. * = significantly higher than in control neurons incubated with αSN.

Since PGE_2_ causes synapse degeneration [16] the effects of drugs that inhibit cyclo-oxygenases (COX), enzymes that convert arachidonic acid to prostaglandins, upon PGE_2_ production in synaptosomes was studied. Pre-treatment of synaptosomes with the COX inhibitors aspirin or ibuprofen significantly reduced the αSN-induced increase in PGE_2_ (Figure 3A). In contrast pre-treatment with drugs that inhibit lipoxygenases (LOX), enzymes that convert arachidonic acid to leucotrienes, (caffeic acid or nordihydroguaiaretic acid (NDGA)) did not affect αSN-induced increase in PGE_2_. Furthermore, pre-treatment of cultured neurons with aspirin or ibuprofen protected neurons against αSN-induced loss of synaptophysin (Figure 3B) or CSP (Figure 3C) whereas pre-treatment with caffeic acid or NDGA had no significant effect.

### 2.4. PAF Antagonists Protected against αSN-Induced Synapse Degeneration

**Figure 4:**
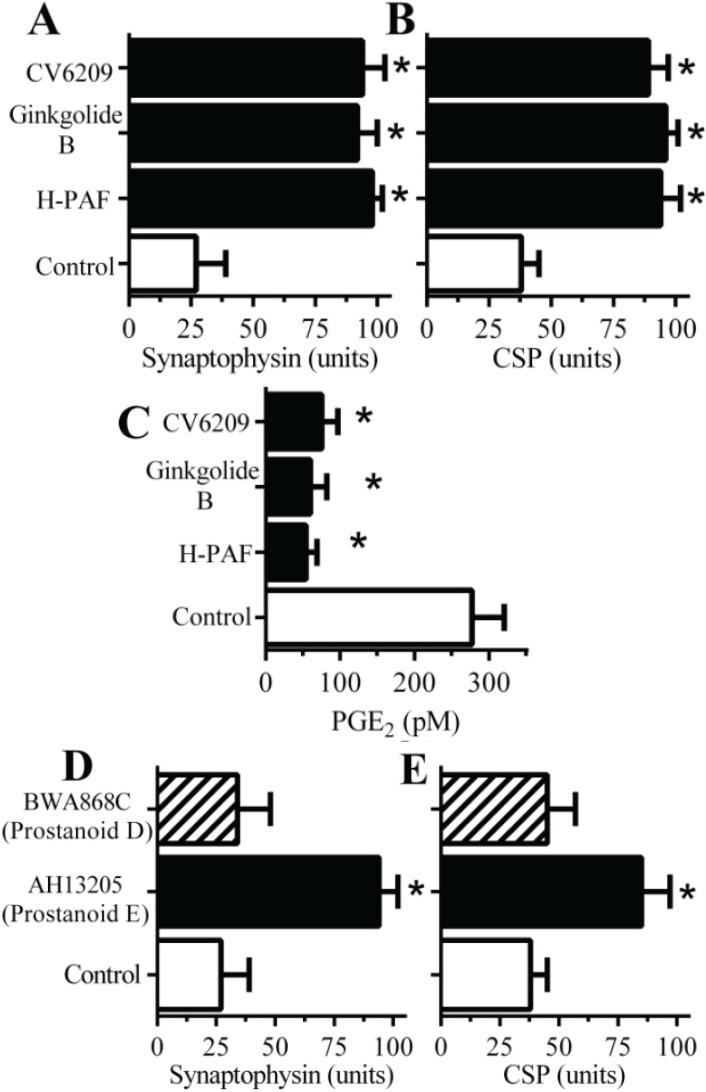
Platelet-activating factor (PAF) antagonists protect neurons against αSN-induced synapse damage—The amounts of synaptophysin (**A**) and CSP (**B**) in neurons pre-treated with a vehicle control (□) or PAF antagonists (1 µM H-PAF, ginkgolide B or CV6209 (■)) and incubated with αSN. *= significantly higher than in control neurons incubated with αSN. Values are means ± SD from triplicate experiments performed four times, *n* = 12; (**C**) The concentrations of PGE_2_ produced by synaptosomes pre-treated with incubated with a vehicle control (□) or PAF antagonists (1 µM H-PAF, ginkgolide B or CV6209 (■)) and incubated with 500 nM αSN. Values are means ± SD from triplicate experiments performed three times, *n* = 9. * = significantly lower than control synaptosomes incubated with 500 nM αSN. The amounts of synaptophysin (**D**) and CSP (**E**) in neurons pre-treated with a vehicle control (□), the prostanoid EP receptor antagonist AH13205 (■) or the prostanoid DP receptor antagonist BWA868C (striped bars) and incubated with 1 μM αSN. Values are means ± SD from triplicate experiments performed four times, *n* = 12. * = significantly higher than in control neurons incubated with αSN.

The activation of PLA_2_ is also the first step in the production of platelet-activating factor (PAF) [20] that has been shown to cause synapse degeneration *in vitro* [21]. The addition of PAF receptor antagonists (1-*O*-Hexadecyl-2-acetyl-*sn*-glycerol-3-phospho-(*N*,*N*,*N*-trimethyl)-hexanolamine (Hexa-PAF), CV6209 or ginkgolide B), in the range 0.1–10 µM, did not affect the amount of synaptophysin or CSP in cortical neurons. However, pre-treatment with 2 µM Hexa-PAF, 2 µM CV6209 or 1 µM ginkgolide B provided protection against αSN-induced loss of synaptophysin (Figure 4A) or CSP (Figure 4B). Prior studies showed that PAF facilitates the production of prostaglandins [22] suggesting that one or more of the prostaglandins produced in response to αSN are responsible for synapse degeneration. Here we show that pre-treatment of synaptosomes with PAF antagonists significantly reduced the αSN-induced production of PGE_2_ by synaptosomes (Figure 4C). Prostaglandin E_2_, acts via specific prostanoid E receptors [23] and pre-treatment with the prostanoid E receptor antagonist AH13205, but not the prostanoid D receptor antagonist BWA868C, prevented the loss of synaptophysin and CSP in cortical neurons incubated with αSN (Figure 4D,E). Collectively, these results show that the effects of αSN on synapses were ultimately mediated through prostanoid E receptors.

### 2.5. αSN-Induced Activation of cPLA_2_ Is Cholesterol Sensitive

**Figure 5:**
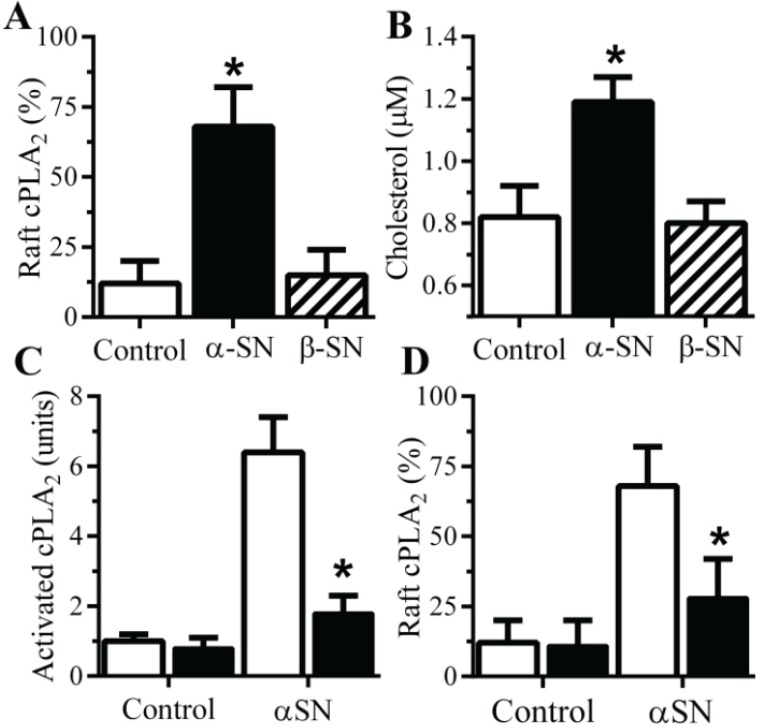
The αSN-induced activation of synaptic cPLA_2_ is sensitive to cholesterol depletion. (**A**) The amounts of cPLA_2_ in rafts derived from synaptosomes incubated with control medium (□) 1 μM αSN (■) or βSN (striped bar). Values are means ± SD from triplicate experiments performed three times, *n* = 9; *=significantly higher than in control synaptosomes. (**B**) The concentrations of cholesterol in synaptosomes incubated with control medium (□) 1 μM αSN (■) or βSN (striped bar). Values are means ± SD from triplicate experiments performed three times, *n* = 9. * = significantly higher than in control synaptosomes. The amounts of activated cPLA_2_ in synaptosomes pre-treated with control medium (□) or 1 μM squalestatin (■) and incubated with 1 μM αSN or PLAP as shown. Values are means ± SD from triplicate experiments performed three times, *n* = 9; * = significantly lower than in control synaptosomes incubate with αSN.(**D**) The amounts of cPLA_2_ in rafts derived from synaptosomes from neurons treated with control medium (□) or 1 μM squalestatin (■) and incubated with 1 μM αSN. Values are means ± SD from triplicate experiments performed three times, *n* = 9. * = significantly lower than in control synaptosomes incubated with αSN.

The activation of cPLA_2_ is associated with its migration to specific membrane micro-domains by an *N*-terminal lipid binding motif [24]. Prior studies demonstrated that Aβ-induced activation of cPLA_2_ in synaptosomes involved its translocation to lipid rafts [19]. Here we show that the addition of αSN, but not βSN, results in the migration of cPLA_2_ to detergent-resistant membranes (rafts) (Figure 5A). Rafts are dependent on cholesterol concentrations and the addition of 1 μM αSN, but not βSN increased cholesterol concentration in synaptosomes (Figure 5B) suggesting that the presence of αSN triggered the formation of a raft in which cPLA_2_ was activated. To explore this hypothesis further the αSN-induced activation of cPLA_2_ in synapses from cholesterol-depleted neurons was examined. Squalestatin is a cholesterol synthesis inhibitor [25] that reduced the cholesterol content of neurons [26]. Treating cultured neurons with 200 nM squalestatin reduced total cellular concentrations without affecting neuronal survival or synapse density in these cultures as measured by synaptophysin (99.2 units ± 3 compared with 100 ± 4, *p* = 0.67) or CSP (97.8 units ± 3.7 compared with 100 ± 4.1, *p* = 0.43). However the αSN-induced activation of cPLA_2_ was significantly reduced in synaptosomes from squalestatin-treated neurons when compared to control synaptosomes (Figure 5C) as was the αSN-induced translocation of cPLA_2_ to rafts (Figure 5D).

### 2.6. The αSN-Induced Synapse Damage Is Cholesterol Sensitive

**Figure 6:**
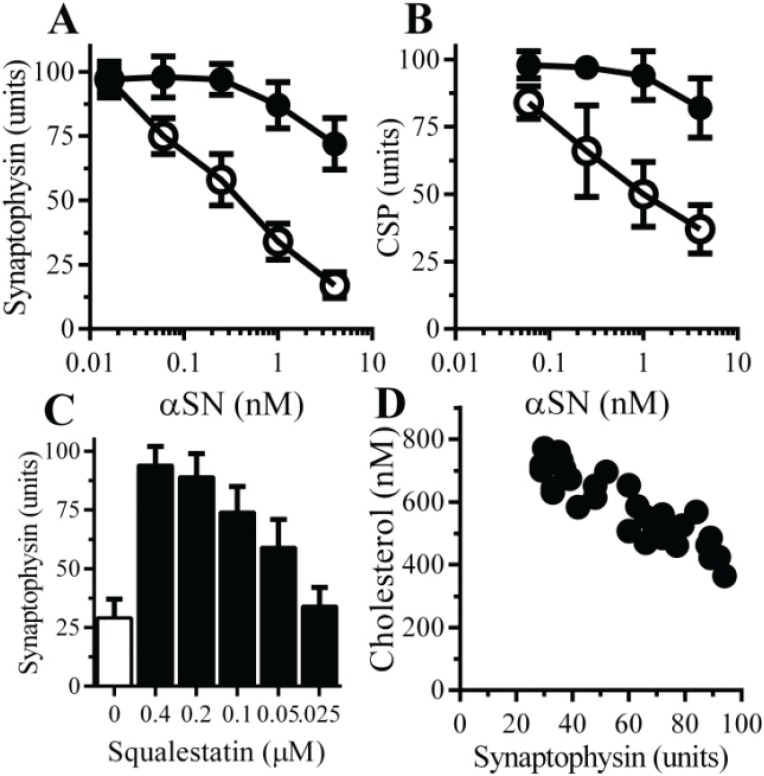
Squalestatin protects neurons against αSN-induced synapse damage—The amounts of synaptophysin (**A**) and CSP (**B**) in neurons pre-treated with control medium (○) or 200 nM squalestatin (●) and incubated with αSN as shown. Values are means ± SD from triplicate experiments performed three times, *n* = 9; (**C**) The amounts of synaptophysin in neurons pre-treated with control medium (□) or squalestatin as shown (■) and incubated with 1 μM aggregated αSN. Values are means ± SD from triplicate experiments performed three times, *n* = 9; (**D**) There was a significant inverse correlation between the concentrations of cholesterol in neurons treated with squalestatin (200 nM to 25 nM) and the amounts of synaptophysin in these neurons after incubation with 1μM aggregated αSN, Pearson’s coefficient = −0.908, *p* < 0.01.

The role of cholesterol in αSN-mediated synapse damage was examined. Pre-treatment with 200 nM squalestatin protected neurons against the αSN-induced loss of synaptophysin (Figure 6A) or CSP (Figure 6B). The efficacy of squalestatin was also tested; pre-treatment with squalestatin produced a dose-dependent increase in the synaptophysin content of neurons subsequently incubated with 1 µM αSN (Figure 6C). The effects of squalestatin on concentrations of cholesterol in neurons were also dose dependent; there were significant correlations between the concentrations of cholesterol and synaptophysin, Pearson’s coefficient = −0.908, *p* < 0.01 (Figure 6D) following the addition of 1 μM αSN.

## 3. Discussion

The present study utilized a pharmacological approach to determine the molecular mechanisms involved in synapse damage induced by aggregated αSN in cultured neurons. We demonstrate that aggregates of αSN, but not β-SN, caused extensive synapse damage, as evidenced by the loss of synaptophysin and CSP, in cultured neurons. Of particular interest were the observations that the concentrations of αSN used in these assays did not reduce levels of caveolin from neuronal cultures and neuronal viability was not affected. We conclude that the *in vitro* assays may be analogous to the processes that occur in areas of the PD/LBD brains where synapse damage occurs without any significant neuronal death.

The unregulated activation of PLA_2_ is thought to be pivotal to the pathogenesis of several neurodegenerative diseases [27,28]. Prior studies showed that cPLA_2_ was highly enriched in synaptosomes and that synapse damage in response to “natural Aβ” and prion-derived peptides was dependent upon the activation of cPLA_2_ [16]. Others have also shown that the activation of cPLA_2_ is a key element in neurodegeneration induced by Aβ peptides [29,30], in experimental Parkinson’s disease [31] and Alzheimer’s disease [32]. Here we show that the addition of aggregated αSN increased the activation of synaptic cPLA_2_ suggesting that αSN-induced activation of cPLA_2_ resident within synapses was responsible for synapse damage. In addition, the αSN-induced synapse damage was reduced by pre-treatment with the cPLA_2_ inhibitors. Collectively, these results indicate that the activation of an endogenous cPLA_2_ is a pivotal event in synapse damage mediated by αSN aggregates.

The activation of PLA_2_ leads to the formation of a number of bioactive factors including prostaglandins, leucotrienes, docosanoids and PAF [20,33]. The fact that PAF antagonists reduced the αSN-induced synapse damage are consistent with reports that PAF causes synapse damage [16,34]. We also note that the PAF antagonist ginkgolide B is a component of the Ginkgo biloba extract that have neuroprotective effects in animal models of Parkinson’s disease [35,36]. Synapse damage induced by αSN aggregates was also reduced in neuronal cultures pre-treated with ibuprofen or aspirin, drugs that inhibit cyclooxygenases. The cyclooxygenases convert arachidonic acid to prostaglandins, including PGE_2_. Although PGE_2_ facilitates memory formation at the synapse PGE_2_ [37], it is thought to be a “goldilocks mediator” and high concentrations cause synapse degeneration [16]. In this study a prostanoid EP receptor antagonists reduced synapse damage in response to αSN consistent with the idea that the persistent activation of cPLA_2_ by αSN results in high concentrations of PAF and PGE_2_ that cause synapse damage.

Disturbances in membrane cholesterol are a common indication of neurodegenerative diseases including Parkinson’s disease [38]. An extensive review of the role of cholesterol in PD and cellular and models of PD by Dolga and colleagues [39] showed that at least one epidemiological study has demonstrated that high total cholesterol concentrations was associated with an increased risk of PD [40]. The role of cholesterol in disease progression is inferred from studies examining the effects of cholesterol synthesis inhibitors. Thus, in transgenic mice overexpressing αSN, αSN aggregation and neuronal degeneration were strongly diminished by treatment with a cholesterol synthesis inhibitor [41]. In humans, the use of statins showed a statistically significant inverse association with PD in some reports [42,43] while other studies have reported no such association [44,45]. The inconsistent results may reflect the variable ability of different statins to penetrate the blood brain barrier. In this study we used the squalene synthetase inhibitor squalestatin, which inhibits cholesterol synthesis without affecting non-sterol products [46] to show that αSN aggregates induced the activation of cPLA_2_ and synapse damage, and that these were cholesterol dependent. The concentrations of cholesterol in cell membranes affects many different cell functions; it is critical for the formation of rafts [47] that act as membrane platforms to concentrate molecules for cell signalling [48] including cPLA_2_ [49,50]. These results are consistent with the hypothesis that cholesterol depletion disrupts the formation of the raft in which αSN activates cPLA_2_.

## 4. Experimental Section

**Primary Neuronal Cultures**—Cortical neurons were prepared from the brains of mouse embryos (day 15.5). Neurons were plated at 2 × 10^5^ cells/well in 48 well plates (coated with 5 μg/mL poly-L-lysine) in Ham’s F12 medium containing 5% foetal calf serum for 2 h. Cultures were shaken (600 r.p.m for 5 min) and non-adherent cells removed by 3 washes in PBS. Neurons were grown in neurobasal medium (NBM) containing B27 components and nerve growth factor (5 nM) for 10 days. Immunohistochemistry showed that the cells were greater than 95% neurofilament positive. Fewer than 3% of cells stained for glial fibrillary acidic protein (astrocytes) or for F4/80 (microglial cells). Neurons were incubated with peptides for 24 h, washed 3 times with PBS and homogenised in a buffer containing 150 mM NaCl, 10 mM Tris-HCl, pH 7.4, 10 mM EDTA, 0.5% Nonidet P-40, 0.5% sodium deoxycholate, 0.2% sodium dodecyl sulphate (SDS) and mixed protease inhibitors (4-(2-Aminoethyl) benzenesulfonyl fluoride hydrochloride (AEBSF), Aprotinin, Leupeptin, Bestain, Pepstatin A and E-46) (Sigma,Poole, UK) and a phosphatase inhibitor cocktail including PP1, PP2A, microcystin LR, cantharidin and p-bromotetramisole (Sigma) at 10^6^ cells/mL. Nuclei and cell debris was removed by low speed centrifugation (300× *g* for 5 min).

**Western Blotting**—Samples were mixed with Laemmli buffer containing β-mercaptoethanol, heated to 95 °C for 5 min and proteins were separated by electrophoresis on 15% polyacrylamide gels (PAGE). Proteins were transferred onto a Hybond-P PVDF membrane by semi-dry blotting. Membranes were blocked using 10% milk powder; synapsin-1 was detected with goat polyclonal (Santa Cruz Biotech, London, UK), vesicle-associated membrane protein (VAMP)-1 with mAb 4H302 (Abcam, Cambridge, UK) and caveolin with rabbit polyclonal antibodies to caveolin Santa Cruz Biotech. Human recombinant αSN was separated by PAGE under non-denaturing conditions and was detected by incubation with mAb 211 raised against amino acids 121 to 125 of human αSN (Santa Cruz Biotech). These were visualized using a combination of biotinylated anti-mouse/goat/rabbit IgG (Sigma), extravidin-peroxidase and enhanced chemiluminescence.

**Synaptosome Preparations**—Synaptosomes were prepared on a discontinuous Percoll gradient [51]. Briefly, 10^6^ cortical neurones were homogenized at 4 °C in 1 mL of SED solution (0.32 M sucrose, 50 mM Tris-HCl, 1 mM EDTA, and 1 mM dithiothreitol, pH 7.4 and mixed protease/phosphates inhibitors (as above)). The preparation was centrifuged at 1000× *g* for 10 min. The supernatant was transferred to a 4-step gradient of 3, 7, 15, and 23% Percoll in SED solution and centrifuged at 16,000× *g* for 30 min at 4 °C. The synaptosomes was collected from the interface of the 15% and 23% Percoll steps, washed twice (16,000× *g* for 30 min at 4 °C) and suspended in extraction buffer (150 mM NaCl, 10 mM Tris-HCl, 10 mM EDTA, 0.2% SDS and mixed protease/phosphatase inhibitors).

**Isolation of DRMs**—These membranes were isolated by their insolubility in non-ionic detergents as described [52]. Briefly, samples were homogenised in an ice-cold buffer containing 1% Triton X-100, 10 mM Tris-HCl, pH 7.2, 150 mM NaCl, 10 mM EDTA and mixed protease inhibitors and nuclei and large fragments were removed by centrifugation (300× *g* for 5 min at 4 °C). The post nuclear supernatant was incubated on ice (4 °C) for 1 h and centrifuged (16,000× *g* for 30 min at 4 °C). The supernatant was reserved as the detergent soluble membrane (DSM) while the insoluble pellet was homogenised in an extraction buffer containing 10 mM Tris-HCL, pH 7.4, 150 mM NaCl, 10 mM EDTA, 0.5% Nonidet P-40, 0.5% sodium deoxycholate, 0.2% SDS and mixed protease inhibitors at 10^6^ cells/mL, centrifuged (10 min at 16,000× *g*) and the soluble material was reserved as the DRM fraction.

**Synaptophysin ELISA**—The amount of synaptophysin in neuronal extracts was measured by ELISA [16]. Briefly, the capture mAb was anti-synaptophysin MAB368 (Millipore). Samples were added for 1 h and bound synaptophysin was detected using rabbit polyclonal anti-synaptophysin (Abcam) followed by a biotinylated anti-rabbit IgG (Dako), extravidin-alkaline phosphatase and 1 mg/mL 4-nitrophenol phosphate. Absorbance was measured on a microplate reader at 405 nm and the synaptophysin content of samples was expressed as units where 100 units was defined as the amount of synaptophysin in untreated neurons.

**CSP ELISA**—Maxisorb immunoplates were coated with a monoclonal antibody (mAb) to CSP (sc-136468, Santa Cruz) and blocked with 5% milk powder. Samples were added and bound CSP was detected using rabbit polyclonal anti-CSP (sc-33154, Santa Cruz) followed by a biotinylated anti-rabbit IgG, extravidin-alkaline phosphatase and 1 mg/mL 4-nitrophenol phosphate solution. Absorbance was measured at 405 nm. Samples were expressed as “units CSP” where 100 units was the amount of CSP in 10^6^ control cells.

**cPLA_2_ ELISA**—The amount of cPLA_2_ in extracts was measured by ELISA as described [53]. Maxisorb immunoplates were coated with 0.5 µg/mL of mouse mAb anti-cPLA_2_ (clone CH-7—Upstate) and blocked with 5% milk powder in PBS + 0.1% tween 20 (PBST). Samples were incubated for 1 h and the amount of bound cPLA_2_ was detected using a goat polyclonal anti-cPLA_2_ (Santa-Cruz Biotech) followed by biotinylated anti-goat IgG, extravidin-alkaline phosphatase and 1 mg/mL pNPP solution. Absorbance was measured at 405 nm and the amount of cPLA_2_ protein expressed in units, 100 units = amount of cPLA_2_ in control preparations.

**Activated cPLA_2_ ELISA**—The activation of cPLA_2_ is accompanied by phosphorylation of the 505 serine residue which creates a unique epitope and can be measured by ELISA [16]. To measure the amount of activated cPLA_2_, an ELISA using a mAb (anti-cPLA_2_, clone CH-7) combined with rabbit polyclonal anti-phospho-cPLA_2_ (Cell Signalling Technology) followed by biotinylated anti-rabbit IgG (Sigma), extravidin-alkaline phosphatase and 1mg/mL pNPP solution. Absorbance was measured on a microplate reader at 405 nm. Results were expressed as “units activated cPLA_2_” (100 units = amount of activated cPLA_2_ in control preparations). The amounts of PGE_2_ in synaptosomes were determined using a competitive enzyme immunoassay kit (Amersham Biotech, Amersham, UK) according to the manufacturer's instructions.

**Cholesterol and Protein Content**—Cellular cholesterol and protein content were determined in cell extracts. Protein concentrations were measured using a micro-BCA protein assay kit (Pierce, Paisley, UK). The amounts of cholesterol were measured using the Amplex Red cholesterol assay kit (Invitrogen, Paisley, UK), according to the manufacturer’s instructions. Briefly, free cholesterol is oxidised by cholesterol oxidase to yield hydrogen peroxide and ketones. The hydrogen peroxide reacts with 10-acetyl-3,7-dihydroxyphenoxazine (Amplex Red Reagent) to produce highly fluorescent resorufin, which is measured by excitation at 550nm and emission detection at 590 nm.

**Peptides**—Recombinant human αSN and βSN were purchased from Sigma. Stock solutions of peptides were stored at 1 mM, thawed on the day of use, diluted in culture medium and sonicated before they were added to neurons.

**Drugs**—Acetyl salicylic acid (aspirin), AH13205, AACOCF_3_, BWA868C, caffeic acid, CV6209, ginkgolide B, Hexa-PAF, α-Methyl-4-(isobutyl)phenylacetic acid (ibuprofen), MAFP, NDGA, U73122 were obtained from Sigma. U73122 and ethyl-18-OCH_3_ were obtained from Biomol. Squalestatin was a gift from GlaxoSmithKline. Stock solutions were dissolved in ethanol or di-methyl sulphoxide (DMSO) and diluted in medium to obtain final working concentrations. Vehicle controls consisted of equivalent dilutions of ethanol or DMSO in culture medium.

**Statistical Analysis**—Differences between treatment groups were determined by Student’s, 2 sample, paired T-tests. For all statistical tests significance was set at the 1% level.

## 5. Conclusions

We conclude that the addition of αSN reduced the synaptophysin and CSP content of cultured neurons in a tissue culture model of synapse damage. The αSN-induced synapse damage was associated with increased activation of cPLA_2_ within synapses. Drugs that protect the synapse provide a rational strategy to treat neurodegenerative diseases. Here we show that cholesterol synthesis inhibitors, cPLA_2_ inhibitors, COX inhibitors and PAF receptor antagonists all protect cultured neurons against αSN-induced synapse damage. Such results suggest that aberrant activation of the cPLA_2_ pathway by αSN leads to synapse damage and suggests that drugs targeting this pathway, and which are able to cross the blood–brain barrier, should be considered for further testing in animal models of PD.
